# Some After-Effects of the Recent Influenza1Read at the meeting of the Central New York Medical Association, Buffalo, N. Y., June 2, 1891.

**Published:** 1891-09

**Authors:** Frank Hamilton Potter

**Affiliations:** Clinical Professor of Laryngology in the Medical Department of the University of Buffalo; Surgeon-in-charge of the Throat Department, Erie County Eye, Ear and Throat Infirmary


Buffalo Medical and Surgical Journal

VOL. XXXI. SEPTEMBER, 1891. No. 2.

### Original Communications.

### SOME AFTER-EFFECTS OF THE RECENT INFLUENZA.[1]

A POSTHUMOUS PAPER.

BY FRANK HAMILTON POTTER, M. D.,

Clinical Professor of Laryngology in the Medical Department of the University of Buffalo; Surgeon-in-charge of the Throat Department, Erie County Eye, Ear and Throat Infirmary.

THE invitation of your president to present a paper at this meeting was accompanied with a request to make it a short one. My remarks, therefore, under the title that has been selected, will be more in the nature of an introduction of the subject than an exhaustive or detailed report.

Whatever may be our views of the nature of the recent influenza, we are sure of certain observations concerning it. It has an epidemic character and impresses itself, sooner or later in its course, upon the air passages. It is not a simple cold, but has a distinct individuality. What this may be, must be determined by further investigation, when a larger experience will give us more complete data upon which to base some general conclusions. At present we are concerned in calling attention to certain results which, either from the violence of the inflammatory processes or from the nature of the disease itself, continue after the disease has disappeared. These results are seen in the general depressed condition of the nervous system; in inflammation in and about the ear, and in subacute inflammatory laryngeal disturbances. Again, those who before having the influenza did not suffer at all from catarrh of the upper air tract, now appear with various forms of that disease. They will at once inform you that their trouble dates from *la grippe.* Others who had some slight trouble before, now exhibit the catarrhal manifestations in

1. Read at the meeting of the Central New York Medical Association, Buffalo, N. Y., June 2, 1891.

forms much more severe. So we can easily see, besides the damage attending the disease itself, that there is a long list of after-effects that require careful treatment.

Let us first consider the disturbances seen in and about the ears. These consist of middle ear inflammatory changes of all degrees. Occasionally, where the inflammation has been especially severe, we also observe changes in the external auditory meatus. One case came under my observation with the history of recurrent attacks of median otitis. They were mild at first, and after each attack the patient thought there would be no more trouble. Finally, a purulent catarrh developed, the membranous tympanum was partially destroyed, the external auditory meatus nearly occluded, and there were symptoms of involvement of the mastoid cells. Happily the result in this case was favorable, but it illustrates very well not only the insidious character of the effects of the influenza upon the ear, but also the danger of neglecting to properly interpret the symptoms when first seen. Another case was seen with an acute catarrhal inflammation of the middle ear, which appeared promptly after the subsidence of the general symptoms of influenza. The disease developed so rapidly as to cause perforation of the drum; it was very persistent, the patient making a slow, though good recovery. Again, several instances of acute catarrhal otitis without perforation have been seen, and also one case of inflammation of the external auditory meatus without involvement of the middle ear. We can thus see that the effects upon the ear may be as severe in this disease as in any of the exanthemata or general febrile diseases. The conditions of the auditory apparatus should be noticed, therefore, in all cases, so that prompt measures can be employed in order to prevent any permanent mischief.

In the nose some interesting conditions are seen. Patients who, previous to the attack, considered themselves free from what they call "catarrh," now begin to complain of some of the symptoms of that many-sided disease. If true that there has been no previous disease, we usually find in the nose the condition described by some authors under the term of simple chronic rhinitis. This is probably one of the very few opportunities we have of studying this condition. It is seldom seen by itself,—that is to say, without hypertrophic changes being also present. It is an inflammation of a mild but persistent type, not involving the connective tissue, and therefore not showing evidences of hypertrophy. It may be, and is so considered by some writers, as the first step in hypertrophic catarrh. It is distinguished by the resistance of the tissues under the probe, and the complete contraction of the tissues under cocaine. Its prominent symptoms are persistent stenosis with copious secretion. It is relieved by antiseptic washes with astringents. In severe cases applications of chromic acid are indicated.

In cases of old standing catarrh with hypertrophic changes, and with or without bony deformity, we find that the influenza has usually produced a marked aggravation of all symptoms. Many cases of this sort that have come under observation the past Winter and Spring, could be cited in illustration of this statement, but too many details of cases are wearisome and consume time; and, besides, my purpose will be served if this proposition will receive the attention it deserves. In the naso-pharynx and pharynx, especially in the former, we find some after-effects that require to be noted. The glandular structure in the naso-pharynx seems stimulated to great activity by this peculiar epidemic, and as a result we have the disease called naso-pharyngeal catarrh. This differs very much from a chronic nasal catarrh on account of the difference in the naso-pharyngeal tissue. Without discussing what this difference is, we may state that inflammatory action here may take one of two directions: either there may be an increase of the glandular structure, or stimulation of the activity of the glands with destruction of the epithelium. In the one case we have so-called adenoid development; in the other an abundant and persistent muco-purulent discharge. In my experience the influenza was followed by the latter form of disease when secondary results were observed in the naso-pharynx. In these cases the nasal tissues were but little effected, the trouble being confined to the pharyngeal vault. They exhibited many of the characteristics of an old catarrhal trouble in this region, and it did not seem correct to give the disorder such a recent date. The patients, however, were reliable, and assured me that previous to the influenza they had been free from any nose or throat disease.

In this condition we would be interested, naturally, in observing whether the tonsils showed any evidence of being also involved. Two such cases were seen. The tonsils could not be said to be acutely inflamed, neither would the local nor constitutional symptoms warrant this. But they were rather subacutely inflamed, if I may so speak. The follicles were filled with secretion, and the tonsils were swollen, red, and tender. There was some difficulty in swallowing, in speaking, and in respiration, though not sufficient to cause any anxiety. One case had some rheumatic history; the other showed no such constitutional disturbance.

In another case edema of the uvula was seen. This was in an infirmary patient in the service of my assistant, Dr. Henry J. Mulford. The patient did not return, so that nothing beyond the fact of the edema being present, and its dependence upon *la grippe* can be given.

Another sequel was the formation of a small abscess in the tissue between the last molar tooth and the tonsil. There was some suspicion of disease of old standing in the tooth, but this was not clearly made out. Two months after recovery I saw the patient, who said the tooth had not been treated up to that time, and had given no trouble since.

I have seen several cases of laryngitis following the influenza. These were attended by loss of voice, usually partial, but in one case almost complete, with pain and cough. The latter was very persistent and of a dry, irritating character. It was difficult to control. In two instances the cough was so severe, and the paroxysms so prolonged, as to greatly exhaust the patients. The larynx was usually found deeply congested, especially along the vocal cords and about the arytenoids. There was not much apparent swelling, but the articulations must have been involved, as any movement caused pain. To the eye also the action of the larynx seemed halting and uncertain.

Nothing has been said about the treatment of the conditions thus passed in review — perhaps altogether too briefly. The purpose has been simply to notice those after-effects that have come under my own observation. For this reason, also, no reference has been made to the literature, already rapidly accumulating, relating to this disease and its effects upon the air passages.

Passing down into the chest we find bronchial and pneumonic complications of the inflammatory type. Pneumonia following *la grippe* usually has been fatal. Cases of this character must have come under the observation of almost everyone present, and reference is made to it merely to complete the list, so far as the writer is concerned, of the after-effects of the influenza as seen in the air tract. There may be other secondary changes, but all those have been mentioned that have fallen within my experience. The list of authors who have called attention to these sequelæ is already large. It would be instructive to compare their views, which may be done on some other occasion.

We can see, however, from this short account, based though it be upon a limited experience, that the influenza, prevalent during the past two years, is prone to leave behind it some disorder of the air tract that is important and may be serious. We conclude, therefore, that the disease should be watched for any of these late manifestations, the patient warned against too early exposure, and informed of the importance of prompt treatment, should any of the above-described complications arise.

